# Feasibility and Cultural Adaptation of a Community-Engaged Physical Activity Intervention for Hispanic Older Adults: Pilot Study

**DOI:** 10.2196/65489

**Published:** 2025-05-27

**Authors:** Zvinka Z Zlatar, Mikael Anne Greenwood-Hickman, Lazaro N Martinez Lujan, Julie Cooper, Stefani Florez-Acevedo, David X Marquez, Rosa Gutierrez Aceves, Andrea Paula Vargas, Dori E Rosenberg

**Affiliations:** 1 Department of Psychiatry, University of California, San Diego, 9500 Gilman Drive MC 0811, La Jolla, CA, 92037, United States, +1 8588227737; 2 Kaiser Permanente Washington Health Research Institute, Seattle, WA, United States; 3 Department of Health Systems and Population Health, School of Public Health, University of Washington, Seattle, WA, United States; 4 Department of Kinesiology and Nutrition, University of Illinois Chicago, Chicago, IL, United States

**Keywords:** lifestyle, dementia risk reduction, brain health, sedentary, exercise

## Abstract

**Background:**

The adult Hispanic population of the United States faces an increased risk of dementia compared to non-Hispanic White adults. Physical activity (PA) can help reduce dementia risk, but culturally adapted interventions for Hispanic populations are lacking. Culturally adapted interventions are needed to increase uptake and adherence to brain health promotion strategies in the Hispanic community.

**Objective:**

The De Pie y a Movernos intervention aims to reduce barriers to participation in clinical research by culturally adapting a remotely based PA intervention for middle-aged and older Hispanic adults and establish its feasibility and acceptability through a pre-post pilot study. Findings from the cultural adaptation process will inform a stage II PA randomized controlled trial.

**Methods:**

The adaptation process followed Barrera and Castro’s 2006 cultural adaptation framework and included a literature review, translation of intervention materials, review by a Hispanic-comprised community advisory board and bilingual staff, and a pre-post pilot study (N=10) with subsequent focus groups to refine the intervention. The pilot intervention included the use of Fitbit activity trackers and 2 individualized goal-setting calls with a health coach over a period of 3 weeks. Feasibility and acceptability were assessed using both quantitative methods and qualitative focus groups. Primary quantitative outcomes included enrollment, recruitment, and completion rates, as well as acceptability (predetermined satisfaction survey scores ≥3). Focus groups were thematically coded to identify themes for participants’ opinions about several aspects of the intervention and explore key barriers and facilitators to PA engagement to improve the planned stage II trial.

**Results:**

Ten Hispanic adults (age: mean 62.7, SD 5.3 years; education: mean 11.8, SD 3.8 years; n=9, 90% female; n=9, 90% Spanish-speaking) participated in the pre-post pilot, with a 100% completion rate, 50% enrollment rate, and a recruitment rate of 5 participants per month. Acceptability was high (mean score 4.6, SD 0.3; range 1‐5). Qualitative analyses indicated that participants had high satisfaction with the intervention. They expressed a preference for adding group-based activities and increased interaction with study staff. Key barriers to PA included lack of awareness about the benefits of PA, low self-efficacy, time constraints, health conditions, and weather, while facilitators included awareness of PA’s cognitive benefits, social support from family or friends, accountability, enjoyable activities, self-efficacy, and Fitbit use. Insights from participants and community advisory board recommendations led to modifications for the larger trial, such as incorporating group-based elements for those who want them and adding an option for teleconference coaching calls.

**Conclusions:**

The cultural adaptation process was essential in refining the intervention to align with the preferences of older Hispanic adults, which resulted in a feasible and acceptable intervention. Findings will inform a planned stage II randomized controlled trial aimed at promoting PA and reducing dementia risk in older Hispanic adults.

## Introduction

Older adults from the Hispanic or Latino community (henceforth referred to as Hispanic) face numerous health disparities [1-4], including higher rates of Alzheimer disease and related dementias (ADRD) [5-7] and cardiometabolic conditions like diabetes, hypertension, and obesity, which are more prevalent in Hispanic populations compared to their non-Hispanic White counterparts [8-13]. Notably, cardiometabolic conditions increase the risk of developing ADRD, which is a growing public health concern [5,14-18]. Projections indicate that by the year 2060, there will be 3.2 million Hispanic older adults living with ADRD [7], highlighting the urgency for targeted and culturally adapted risk reduction strategies to promote cognition and brain health for this population.

The 2024 Lancet Commission on Dementia Prevention highlighted that addressing 14 modifiable risk factors, including physical inactivity, could account for up to 45% of dementia cases worldwide [19]. Regular physical activity (PA) is associated with improved cognitive function [20,21], reduced neurodegeneration [22], higher cerebral blood flow [23], and a lower risk of ADRD [24,25]. However, older Hispanic adults engage in PA at significantly lower rates than their White counterparts [26] in part due to institutional (less information or education about PA, financial constraints, and lower access to health care), societal (caregiving responsibilities, lack of childcare, and poor spousal support), and environmental barriers (weather, less access to green spaces, facilities, or safe places to exercise) [27,28]. These disparities highlight the need to develop novel and culturally adapted PA interventions that address these barriers and support engagement in PA among older Hispanic adults to improve brain health. Using community-engaged approaches such as cultural adaptation, involving a community advisory board (CAB), and including bicultural/bilingual staff can increase adherence and satisfaction by making PA and other lifestyle behavior interventions more culturally relevant, acceptable, and accessible to the target communities [29-34].

To address this gap, we developed the culturally adapted De Pie y a Movernos intervention, a fully remote and individualized PA intervention designed to gradually increase PA to reduce ADRD risk among middle-aged and older Hispanic adults. This manuscript describes the cultural adaptation process informed by community engagement strategies [35] and presents feasibility and acceptability outcomes from a brief pre-post pilot study and follow-up participant focus groups. We also summarize qualitative findings about participants’ opinions of the study as well as perceived barriers and facilitators. Findings from this pilot trial will inform the development of a larger stage II randomized controlled trial (RCT) to determine the efficacy of the De Pie y a Movernos intervention to increase objectively measured PA while investigating changes in mechanisms of adherence and cognitive function.

## Methods

### Study Description

**Figure 1.**
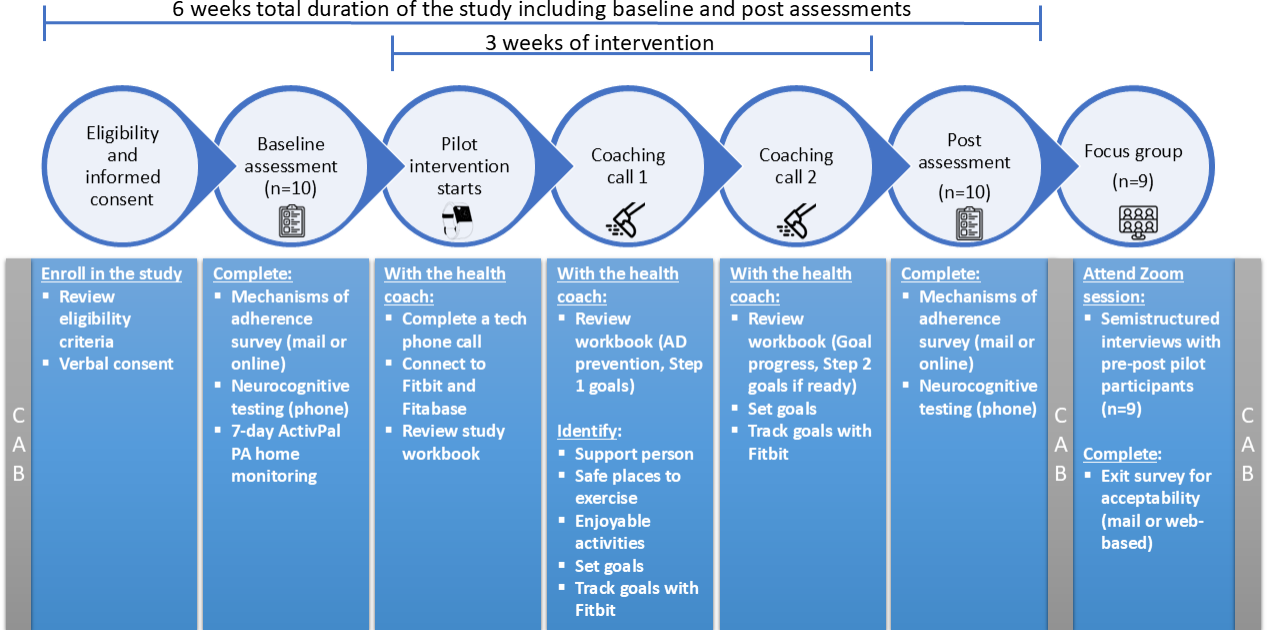
Depiction of the De Pie y a Movernos pilot study timeline and main procedures. The study lasted 6 weeks in total when taking into consideration the baseline and post assessments. The De Pie y a Movernos intervention itself lasted 3 weeks and included a tech phone call to set up the activity tracker at week 1 (Fitbit Inspire 2), followed by 2 phone calls from a health coach at weeks 2 and 3 of the intervention (N=10). Focus groups were conducted after the postintervention assessments (n=9). Nine out of the 10 participants who completed the intervention participated in the focus groups. AD=Alzheimer disease; CAB: community advisory board*;* PA: physical activity.

The goal of the De Pie y a Movernos study was to culturally adapt a PA intervention for the older and middle-aged Hispanic community based on participant and community feedback gathered during a short pre-post pilot study, with the ultimate goal to later test the adapted intervention in a larger, fully remote, stage II RCT. The pre-post pilot intervention lasted 3 weeks and was comprised of 2 phone calls with the study health coach. The Fitbit Inspire 2, a commercially available activity tracker with heart rate monitoring, was used as an intervention tool that helped the health coach guide individualized PA goals. See Figure 1 for a timeline of the study and its components.

### Cultural Adaptation Process

#### Overview

We followed the Barrera and Castro four-phased cultural adaptation approach [36,37]: (1) information gathering, (2) preliminary adaptation design, (3) preliminary adaptation tests, and (4) adaptation refinement. We approached each step in a community-engaged way [35] and considered several cultural dimensions underscored by Bernal et al [38] (eg, language, study team members from similar cultural backgrounds, incorporating cultural values and activities).

#### Information Gathering

We reviewed the literature for existing PA interventions developed for older Hispanic adults and convened a CAB comprised of members of the target population. The CAB was carefully crafted to represent middle-aged and older Hispanic adults with individuals from different sociodemographic backgrounds. The CAB was comprised of 8 members from different countries, all bicultural and some bilingual (with several monolingual Spanish speakers). One of the members is a PhD-level scientist with years of experience developing PA interventions for older Hispanic adults, 4 members are community health workers (promotores de salud) working with Hispanic older adults and Hispanic organizations, 1 member is involved in public health and has extensive experience working with the Hispanic community, another member has expertise in linguistics, and 1 member is a community-dwelling retired bicultural adult. Furthermore, the De Pie y a Movernos study team, which provided further feedback about translations, materials, and cultural relevance, was comprised of bilingual and bicultural faculty (PhD-level) and staff (graduate students, research assistants, and a community health worker) from Chile, Mexico, and Colombia.

#### Preliminary Adaptation Design

Based on findings and input from the information-gathering phase, we customized recruitment strategies and materials for the Hispanic community. The CAB met with the study team prior to conducting the pre-post pilot intervention, and all members of the CAB were offered a Fitbit Inspire 2 so they may provide feedback regarding the use and acceptability of the Fitbit as an intervention tool. The CAB helped the study team develop the study name and logo, review recruitment flyers/brochures, devise a suitable recruitment strategy, and review other participant-facing materials to ensure they were relevant to the Hispanic population.

#### Preliminary Adaptation Tests

We conducted a 3-week pre-post pilot study of the De Pie y a Movernos intervention designed and preliminarily adapted following the steps described above. Details of the pilot study design are outlined in the following section, “Intervention Pilot Study.”

#### Adaptation Refinement

Following the pre-post pilot study, we assessed feasibility and acceptability using a mixed methods approach through follow-up surveys, analysis of pilot intervention study data, and 2 focus groups with all pilot participants focused on gathering feedback about the intervention. We additionally conducted a debrief meeting with the CAB where these findings were presented, and additional adaptation feedback was shared with the study team. Details on feasibility and acceptability assessment, including focus group and qualitative analysis methods, can be found in the “Feasibility and Acceptability Measures” section.

### Intervention Pilot Study

#### Overview

All participants were recruited between March 21, 2023, and May 26, 2023, from the San Diego County community and surrounding regions in Southern California following recommendations from the CAB. The members of the CAB provided ideas of places that could be targeted and input regarding the study pamphlet and flyers. Community outreach was conducted via our trained promotora de investigación (community research worker), who has established relationships with several community partners, agencies, and other community health worker networks that are knowledgeable of the Hispanic community of San Diego County. Our community research worker attended events such as health fairs and education sessions at community centers which are organized by several community organizations. During these community-facing events, the study community research worker recruited participants directly from the community.

#### Inclusion and Exclusion Criteria

Individuals ages 55‐89 years who self-identified as Hispanic and who were able to have a phone conversation, able to walk 1 block unassisted, had no planned surgeries or travel that would interfere with participation for their 6 weeks in the study, were able to speak and read Spanish or English, were willing to wear a Fitbit and activPAL accelerometer, and had a smartphone were eligible to participate. Individuals who self-reported more than 90 minutes per week of PA, less than 6 hours per day sitting, employment in a physically active or standing job, a fall that resulted in hospitalization in the past year, having been told by a doctor they should not engage in PA, a new cancer diagnosis in the past year, or a myocardial infarction or arrhythmia in the past year were excluded. Additionally, individuals were ineligible if they scored ≤4 on the 6-item screener [39] administered by study staff at the initial screening call. Those who self-reported having a dementia diagnosis were also excluded.

#### Intervention Design

**Figure 2.**
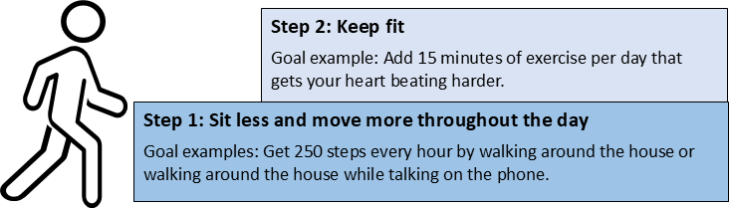
Conceptual model to help participants gradually move toward moderate intensity activity by sitting less and moving more throughout the day. This conceptual model guided the intervention coaching calls, which were individualized for each participant.

The pilot intervention was based on social cognitive theory and habit formation theory [40,41] and targeted the promotion of self-efficacy (gradually building behavior from sitting less and moving more to staying fit), habit strength (creating strong connections between existing daily habits and new PA behaviors), social support (finding a “buddy” to help achieve PA goals and increase accountability), and enjoyment of PA (finding individualized activities that are enjoyable for each participant to sustain adherence). To support these mechanisms, this pilot used a modified “staircase approach” [42] as seen in Figure 2. Step 1 involved focusing on sitting less and moving more throughout the day. Once participants felt ready, they could move to step 2, which encouraged structured moderate-intensity PA. Additionally, participants received a Fitbit Inspire 2 to help them track their progress and to inform goal setting. All participant Fitbits were connected to the Fitabase platform, a research-focused data management platform for Fitbit and other wearable devices [43], which allowed the health coach to access participant data to inform coaching and goal setting. The abbreviated intervention was administered over 3 weeks and consisted of a tech check-in call, where the coach provided technical assistance with the Fitbit device and Fitabase connection, and 2 coaching sessions focused on reviewing study workbook content and setting progressive step 1 and, if the participant was ready, step 2 goals.

#### Health Screening

Based on guidance from the project’s safety officer, who is a practicing physician, participants were screened for safety to engage in PA using the Exercise and Screening for You screening tool [44]. Only participants who answered “no” to all items were allowed to set goals for step 2 (keeping fit; see Figure 2). Participants who answered “yes” to any of the questions were encouraged to set step 1 goals only.

### Ethical Considerations

This study was reviewed and approved by the Kaiser Permanente - Interregional Institutional Review Board (IRB) as the IRB of record. The IRB at the University of California, San Diego, relied on the IRB of record (UCSD IRB: #806367; KPiIRB #1987547-1). Because this study was carried out via telephone, the IRB approved a waiver of signed informed consent. All participants received a study information sheet via mail or email which was discussed with study staff via telephone. After a full explanation of all study procedures and allowing plenty of time for participants to ask questions about the study, participants provided verbal consent to formally enroll in the study. All data were kept confidential and used only for study purposes. All participants were assigned a study ID for the data being collected. All identifiable data, such as contact information, were safely stored using Research Electronic Data Capture (REDCap) managed by Kaiser Permanente Washington Health Research Institute. REDCap is a password-protected and Health Insurance Portability and Accountability Act**–**compliant platform heavily used in research. As compensation for their participation in the pre-post intervention pilot study, participants received US $40 in cash. Additionally, they received US $50 for completing the follow-up focus group and kept the Fitbit Inspire 2 band (approximately US $100 in value).

### Feasibility and Acceptability Measures

We used a mixed methods approach to assess feasibility and acceptability and to inform needed refinements of the culturally adapted intervention program. Below we detail both the qualitative and quantitative methods we used in this study.

#### Qualitative Measures and Analysis

Qualitative feedback on program acceptability and adaptations needed were collected via focus group with participants who completed the pre-post pilot intervention. All pilot intervention participants were invited to participate in a focus group discussion as part of their overall study participation. After completing the intervention, participants confirmed their interest in attending the focus group session during their final measurement phone call. Based on extensive input from all members of the study team, we developed a semistructured focus group discussion guide, a written document that included key questions and possible prompts. To inform ongoing cultural adaptation and refinement, our goal in this discussion was to elicit feedback about participant experiences with the De Pie y a Movernos pilot intervention program and how it resonated with their lives. Discussion topics included (1) aspects of the intervention or study measurement activities participants most enjoyed, (2) parts of the intervention or measurements participants disliked or would change, (3) reactions to completing study activities remotely by phone, (4) interest in and comfort with using video platforms for study activities, (5) barriers to participating in the intervention or study measurement activities, (6) types of physical activity participants most enjoy and resonate with, (7) reactions to the stair step structure (Figure 2) of the intervention, and (8) participants motivators to participate in the study. The final discussion guide is available in Multimedia Appendix 1.

Two study team members (ZZZ and SF-A) cofacilitated two 2-hour digital focus group sessions using the Zoom platform. ZZZ was a multiple principal investigator on the study and a bilingual Hispanic woman from Chile, trained in neuropsychology (PhD). SF-A was the primary measurement staff member and bilingual Hispanic woman from Colombia, trained in psychology and health services research. Based on participant preference, both focus groups were conducted in Spanish, though a separate English session was offered. Two additional study team members observed each focus group discussion, and team members kept informal notes during the focus group discussion to inform team debrief discussions.

Thematic analysis followed the recursive process outlined by Braun and Clarke [45,46], which consists of six key steps: (1) familiarization with the data, (2) code generation, (3) constructing themes from related codes, (4) reviewing and refining themes, (5) describing each theme, and (6) writing an analytic narrative. Two team members, bilingual in Spanish and English, reviewed translated transcripts along with audio recordings to ensure retained meaning. Each member of a 3-person coding team (the Hispanic woman from Colombia described earlier [SF-A], a Hispanic man from Mexico trained in medicine [LML], and a non-Hispanic woman from the United States [MAG-H] trained in anthropology and public health) carefully reviewed translated transcripts and independently coded each transcript (step 1). Using a primarily deductive approach to thematic analysis [45,47], we developed an initial code list informed by focus group questions. Codes were iteratively refined during coding and reconciliation by the coding team (final codebook available in Multimedia Appendix 2). After 1 round of independent coding, the coders met to reconcile discrepancies, refine code definitions, and assign final codes (step 2). Data from each code were reviewed, and themes were collaboratively constructed and refined by the coding team (steps 3 and 4). Final themes were each summarized along with supporting quotations in a summary table, and a summary of findings was presented to the larger study team and CAB (steps 5 and 6). Due to the small volume of data, qualitative coding was managed using the comment and search functions in Microsoft Word. We followed the Consolidated Criteria for Reporting Qualitative Research (COREQ) guidelines for reporting our qualitative methods and findings [48].

#### Quantitative Measures

##### Overview

We developed a study satisfaction survey in-house to obtain quantitative data related to participant’s opinions of the pre-post pilot intervention. The satisfaction survey was collected at the end of the study and before the focus groups. All questions were scored on a Likert-type scale from 1=strongly disagree to 5=strongly agree, and covered satisfaction with study logistics (phone contacts and alternative contact modes), study measurement devices (Fitbit Inspire 2 and activPAL), measurement procedures, clarity of study materials, and the intervention’s relevance to participants’ lives. Example questions included (1) “Overall, I liked the study and found it helpful,” (2) “The Fitbit device was helpful and easy to use,” and (3) “I liked that I didn’t have to travel anywhere for study activities and could participate by phone and mail.” The complete satisfaction survey is available in Multimedia Appendix 3. For each item, an average score ≥3 (neutral, agree, or strongly agree) was the threshold for acceptability.

##### Feasibility

The recruitment rate was defined as the total number of participants recruited divided by the total number of months the trial was actively recruiting. The enrollment rate was defined as the number of enrolled participants divided by the number of prescreened participants x 100. Completion rate was defined as the number of participants who completed the intervention divided by the number of participants who enrolled in the intervention x 100. Furthermore, to assess the feasibility of collecting key measures of interest in our target population, we collected a full measurement battery (objective neurocognitive testing, accelerometer assessment, and mechanisms of adherence) from all pilot participants at the baseline session and after the intervention was completed. Successful completion of these measures was considered an indicator of feasibility. We present descriptive statistics to demonstrate the successful collection and completion of these assessments. We hypothesized that any change in these measures would trend toward increased PA, promotion of mechanisms of adherence, and maintenance of cognition. However, we did not expect significant intervention effects on these outcomes of interest in the abbreviated 3-week pre-post pilot intervention period.

##### PA Assessment

We collected activPAL accelerometer data at baseline only to test the feasibility of deploying research-grade accelerometers in the community. The activPAL is a very small and lightweight inclinometer [49], which is waterproofed in a plastic casing and adhered to participants’ thighs using medical-grade adhesive. To facilitate the appropriate application and wearing of the activPAL, study staff checked in with participants and provided guidance during the tech call described previously. activPAL is valid for measuring steps and sitting time [50,51] and has been used in studies with Hispanic populations [52]. Participants wore the device for 7 days and kept a sleep log. Data were processed in PALanalysis using activPAL’s VANE algorithm to estimate daily steps, standing time (minutes/day), stepping time (minutes/day) and sit-to-stand transitions averaged over the number of wear days.

Additionally, data from each participant’s Fitbit Inspire 2 were extracted using the “Daily Activities” file from the Fitabase platform. Average daily steps, lightly active minutes, fairly or very active minutes, and sedentary minutes were calculated across all days in the first week after their completed tech call (baseline) and during week 6 after the coaching call (post intervention). Because we had access only to daily summary-level Fitbit data, it was not possible to identify and remove individual periods of device nonwear. To approximate this using the available summary data, the accumulation of fewer than 500 recorded steps in a calendar day was considered an indication that the device was not worn for a significant portion of the day. In these instances, the summary data from that day were considered invalid and removed from the analytic sample. Notably, the Fitbit was provided to participants after their baseline activPAL wear. Therefore, due to differences in when participants received and were instructed to wear each device, the summarized baseline data for the Fitbit did not align with the week of baseline activPAL wear, and the 2 summaries should not be directly compared.

##### Mechanisms of Adherence

Hypothesized mechanisms of adherence were measured at baseline and postintervention via REDCap electronic survey unless participants requested the materials be mailed to them. All mechanisms of adherence were assessed with scales that have validated and comparable Spanish and English versions [53-56] and were assessed as point-in-time measures that reflect the individual’s state at the time of assessment. All assessments were conducted in the participant’s language of preference. The Self-Efficacy for PA questionnaire is a 5-item scale (1=not confident and 5=extremely confident) that assesses confidence for engaging in PA in different contexts [54]. The Self-Report Habit Index is a 12-item index that assesses habit-building strength on a scale from 1 to 5 (1=never and 5=always) [55]. The Social Support for Exercise Scale has 3 subscales that assess support from family (10 items) and friends (10 items), and family rewards or punishments (3 items) around engaging in PA with responses ranging from 1=none to 5=very often [53]. The Physical Activity Enjoyment Scale consists of 16 items (1=totally disagree and 5=totally agree) that measure the enjoyment of PA [56*].*

##### Objective Neurocognition

Objective neurocognition was measured via telephone with the following subtests from the National Alzheimer’s Coordinating Center Uniform Data Set version 3 neuropsychological assessment [57]: Craft story 21 Immediate and Delayed Recall, Number Span, Category Fluency, and Phonemic Verbal Fluency. The Uniform Data Set was translated into Spanish with cultural relevance in mind and linguistic equivalence to the English versions [58]. We also collected the Brief-Spanish English Verbal Learning Test as a detailed measure of verbal episodic memory available in English and Spanish [59].

## Results

### Participant Characteristics

A total of 20 participants expressed interest in the study and scheduled a screening call. Four individuals declined participation prior to screening, 5 were determined ineligible for screening, and 1 completed screening but withdrew prior to baseline assessment. Participants were between the ages of 55 and 70 years, 90% (n=9) were women, 90% preferred Spanish (n=9), and they had a mean education of 11.8 (SD 3.8) years (Table 1).

**Table 1.** Pre-post pilot participant characteristics (N=10).

Characteristics	Values
Age (years)	
Mean (SD)	62.7 (5.3)
Range	55‐70
Sex (female), n (%)	9 (90)
Education (years)^a^	
Mean (SD)	11.8 (3.8)
Range	6‐17
Race^a^, n (%)	
White or Caucasian	5 (50)
Other	1 (10)
More than 1 race	3 (30)
Employment status^a^, n (%)	
Retired	4 (40)
Employed part-time	1 (10)
Self-employed part-time	1 (10)
Other	3 (30)
Marital status, n (%)	
Married	6 (60)
Divorced	1 (10)
Separated	2 (20)
Widowed	1 (10)
Ethnic identity (multiple categories permitted), n (%)	
Hispanic or Latina	10 (100)
Mexican or Mexican American	9 (90)
No origin shared	1 (10)
Diabetes, n (%)	4 (40)
Hypertension, n (%)	4 (40)
High cholesterol^a^, n (%)	5 (50)
Fall in the last 12 months, n (%)	1 (10)
Smoking, n (%)	
Ever smoked	4 (40)
Current Smoker	0 (0)

a Some values are missing from the participant characteristics table due to participant nonresponse: education (n=2); race (n=1); employment status (n=1), and high cholesterol (n=1). All health indicators are based on participant self-report during the baseline screening phone call.

### Qualitative Measures Findings

#### Overview

A total of 9 out of 10 participants who completed the De Pie y a Movernos pilot study participated in a focus group session in July 2023 (session 1: n=2; session 2: n=7). Only 1 participant did not attend due to scheduling conflicts. Below, we summarize the key participant feedback and themes that arose in the group discussion. A summary table of key themes and feedback, along with supporting quotations, can be found in Multimedia Appendix 4.

#### PA Devices

Participants found that using the Fitbit Inspire 2 was informative, motivating, easy, and enjoyable, with some challenges associated with setting it up, skin irritation, and connection difficulties. Having available tech support phone calls was identified as helpful. The participants identified that having instructional videos for the Fitbit would be helpful. Regarding the activPAL, many participants thought that it was easy to wear, whereas others identified that the device did not work as intended (did not record their data) and that it irritated the skin. There was confusion about whether the activPAL was continuously recording and sending data to the study team.

#### Study Materials

Study materials were found to be relevant and helpful. Participants thought the materials made program goals clear and easy to connect with. However, participants suggested that our materials could be more organized with a clear order for the participants to follow seamlessly. They also described that it would be helpful to present the same information in multiple learning formats to accommodate varying learning styles (eg, narrative and pictures or videos). Participants liked getting to know the study team through the printed biographies and photographs provided to them (which were mailed or emailed to them before their baseline session). Participants wanted more information about cognitive health broadly and its relationship with PA.

#### Study Activities

Participants expressed that the health coaching calls were helpful in setting goals, generating ideas, breaking down content into achievable units, and allowing them to ask questions and receive support. They also identified that the coaching calls were helpful in providing accountability and follow-up on their goals. Participants were satisfied with the amount and content of communication with the study team. They expressed that the communications with the study team made it clear that they cared about the Hispanic culture and provided support, and some participants appreciated that the program was carried out via telephone as a low-tech option that is accessible to them. Some participants expressed the desire for videoconferencing options for the coaching calls and other aspects of the study. Furthermore, there was a strong desire for more in-person opportunities to connect with the community and meet other study participants.

#### Program Structure and Purpose

The goal of the program to develop interventions to reduce the risk of ADRD in Hispanics resonated with participants. This made them feel appreciated as members of the Hispanic community. Moreover, they found that this focus on ADRD risk reduction in Hispanics was important to the Hispanic community. They appreciated that the program was available in Spanish and that the team members were bilingual or bicultural and well acquainted with the Hispanic culture.

#### Barriers and Facilitators to P**A**

While focus group discussion focused primarily on feedback on the study materials, tools and procedures, barriers and facilitators to PA engagement surfaced incidentally during discussion. These are summarized along with supporting quotations in Multimedia Appendix 4. However, because we did not design our sampling framework or discussion guide to explore these topics, it is probable that additional barriers and facilitators exist for this community that were not discussed. Overall, reported barriers to PA, in general, included a lack of awareness about its health benefits, low self-efficacy, health and weather issues, lack of enjoyment, insufficient social support, ingrained sedentary habits, finding the Fitbit annoying or troublesome, and limited time availability. Facilitators of PA engagement in general included increased awareness of its cognitive and physical health benefits, a sense of accountability through study monitoring, improved self-efficacy in one’s ability to be active, and the need for self-care. Enjoyment of activities, social support from friends or family, habit formation, positive experiences with Fitbit, and perceived improvements in physical and mental health were also discussed as facilitators of PA engagement.

### Quantitative Measures Findings

#### Acceptability

**Figure 3.**
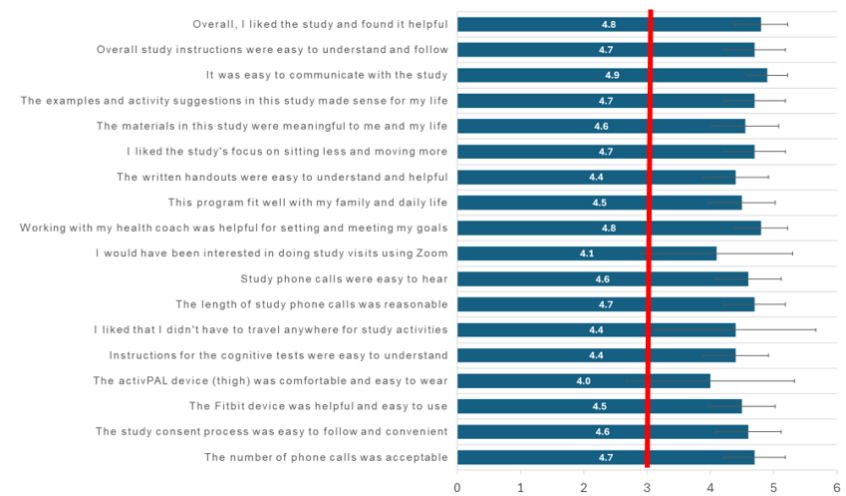
Participant satisfaction survey. Responses to the satisfaction survey are based on feedback from 7 participants. There was missingness from participant nonresponse on the following items: activPAL satisfaction (n=1) and materials were meaningful (n=1). Error bars denote the SD. The vertical red line indicates the threshold score (mean=3) for an item to be considered acceptable to participants.

The satisfaction survey was completed by 7 participants. Figure 3 shows the participant endorsement of each survey item (all items included in the satisfaction survey are listed on the y-axis of Figure 3). The threshold of acceptability was met for all items (scores≥3) and the average score was 4.6 (SD 0.3). The score for the survey item “There were too many phone calls” (Multimedia Appendix 3) was reversed and renamed since it was the only item for which a higher score would indicate less acceptability. In Figure 3, the reversed and renamed item is listed as: “The number of phone calls was acceptable.”

#### Feasibility

A total of 10 out of 20 prescreened Hispanic adults were eligible and consented to participate, yielding an enrollment rate of 50%. Recruitment took place between March 21, 2023, and May 26, 2023, yielding a recruitment rate of 5 participants per month. All participants who enrolled in the intervention completed the 3-week intervention, leading to a completion rate of 100%. All participants who completed a baseline assessment also completed the follow-up assessment (some missing items as per Table 2). We successfully obtained questionnaire data via REDCap survey or via mail from all 10 participants (9 in Spanish and 1 in English). We also successfully carried out telephone coaching calls without any complaints of hearing difficulties. Seven out of 10 participants were able to successfully complete the baseline activPAL measurement and return valid data. Missing activPAL data were due to device failure (n=1), mail loss (n=1), and participant nonwear (n=1). All participants were able to successfully install the Fitbit app on their smartphones and link their accounts to the Fitabase platform for the health coach to access the Fitbit data. Some participants needed substantial technology support from study staff and technology-savvy family members to set up their accounts, but they were all able to successfully complete this step. Table 2 shows a summary of pre-post means and SDs from the measures we collected at each assessment session. Although we did not expect any changes in our measures of interest given the short pilot intervention of 3 weeks, mean scores trended toward improvement for most neurocognitive tests, enjoyment of PA, social support for exercise, habit strength, and self-efficacy scores increased from pre- to postintervention (not tested for statistical significance given the focus on feasibility metrics and not effectiveness).

**Table 2.** Descriptive statistics for all the measures of interest (N=10). All measures were collected at baseline and postintervention (after 3 weeks), except for the activPAL which was only collected at baseline. These measures were not collected to determine efficacy of the intervention but rather to establish the feasibility of completing these assessments for a planned stage II randomized controlled trial.^a^

	Baseline, mean (SD)	Postintervention, mean (SD)
Fitbit metrics		
Steps/day	7872 (3723)	6932 (2531)
Lightly active minutes/day	267 (125)	245 (90)
Fairly or very active minutes/day	275 (128)	250 (90)
Sedentary minutes/day	747 (340)	804 (237)
activPAL measures^b^		
Steps/day	5184 (1379)	—^c^
Standing time (min/day)	319.5 (155.5)	—
Stepping time (min/day)	71.1 (20.4)	—
Sit-to-stand transitions	46 (8)	—
Mechanisms of adherence		
Physical activity enjoyment^d^	60.0 (15.2)	69.7 (9.7)
Social support for exercise^e^	18.5 (7.7)	26.8 (9.4)
Habit strength^f^	2.3 (0.5)	2.9 (0.3)
Self-efficacy for physical activity^g^	8.4 (2.5)	12.2 (3.8)
Objective neurocognition (National Alzheimer’s Coordinating Center Uniform Data Set version 3)		
Craft Story 21 Recall Immediate – Verbatim^h^	15.7 (6.4)	20.9 (8.8)
Craft Story 21 Recall Immediate – Paraphrase^i^	6.3 (5.3)	5.4 (2.4)
Craft Story 21 Recall Delay – Verbatim^h^	13.8 (6.4)	18.8 (9.6)
Craft Story 21 Recall Delay – Paraphrase^i^	7.1 (5.1)	6.2 (3.0)
Verbal Fluency Total F and L words (P and M in Spanish)^j^	20.1 (4.8)	23.1 (8.4)
Animals^j^	19.0 (4.8)	19.3 (4.7)
Vegetables^j^	12.8 (4.2)	14.8 (3.5)
Number span test forward total correct sequences^k^	5.5 (2.0)	6.1 (1.9)
Number span test backward total correct sequences^k^	5.2 (1.5)	6.3 (1.3)
B-SEVLT trial 1^l^	7.7 (2.9)	9.1 (1.9)
B-SEVLT trial 2^l^	10.0 (2.4)	11.7 (1.9)
B-SEVLT trial 3^l^	11.7 (1.7)	13.0 (1.4)
B-SEVLT recall^l^	10.0 (2.6)	12.2 (2.6)

a Data missing due to participant nonresponse for the following items: baseline—physical activity enjoyment (n=2); postintervention—physical activity enjoyment (n=1), social support for exercise (n=2), self-efficacy for physical activity (n=1), and physical activity habit strength (n=1).

b Based on 7 participants who completed an activPAL wear with at least 6 days of valid data. Collected only at baseline.

c Not applicable.

d Range 16‐80, higher scores indicate more enjoyment of physical activity.

e Range 10‐50, higher scores indicate more social support for exercise from friends and family.

f Range 1‐5, higher scores indicate stronger habit strength for physical activity.

g Range 5‐25, higher scores indicate higher self-efficacy for physical activity.

h Range 0‐44, higher scores indicate more story information was recalled verbatim.

i Range 0‐25, higher scores indicate more story information was recalled while paraphrasing.

j Score represents the number of correct items provided within 1 minute for each letter and category. Higher numbers indicate greater verbal fluency.

k Range 0‐14, higher scores indicate better performance

l B-SEVLT: Brief Spanish-English Verbal Learning Test; range 0‐15 for each trial.

## Discussion

We successfully developed and culturally adapted the De Pie y a Movernos intervention, based on established theoretical frameworks and using community-engaged strategies to determine feasibility and acceptability outcomes from a brief pre-post pilot intervention with 10 Hispanic older adults. Both qualitative and quantitative data indicated that the De Pie y a Movernos intervention was feasible and acceptable to participants. Regarding feasibility, there was a completion rate of 100%, an enrollment rate of 50%, and a recruitment rate of 5 participants per month. Acceptability criteria were well exceeded (mean 4.6, SD 0.3), with all participants rating the intervention as satisfactory (defined as scores≥3 on the satisfaction survey). Qualitative analyses indicated that participants overall had high satisfaction with the intervention. They expressed a preference for adding group-based activities and increased interaction with study staff. Some of the key barriers to performing PA generally included time constraints, health conditions, and weather, while facilitators included awareness of PA’s cognitive benefits, social support from family or friends, accountability, enjoyable activities, self-efficacy, and Fitbit use.

Many elements of the study were well-liked by participants and will be kept in the next iteration, while some adaptations will be made to the next iteration based on feedback from the CAB and participants. These include new training videos (in English and Spanish) about how to set up technologies used in the study like the Fitbit, which will be shared via text message or email (depending on preference). Since older Hispanic adults may face challenges in adopting some technologies due to limited access, digital literacy, and language barriers [60,61], we will also encourage participants to seek help from a loved one or family member (partner, children, friends, and caregivers) to help them set up their Fitbit prior to reaching out to the study team for further assistance. Having a loved one support the technological aspects of the study, while the team provides layers of support, from written instructions to instructional videos in the participant’s language of preference, should help reduce the technological barriers related to using the Fitbit.

Participants also reported wanting more contact with the other study participants as a group. Group sessions were not feasible because this study was designed to be fully remote to reduce transportation barriers (ie, lack of access to reliable transportation or long distances to research facilities) that are commonly faced by the Hispanic community [62,63], which limit their ability to participate in clinical research [64], exacerbating health disparities [65,66]. The remote format rather than a group setting is also more conducive to accommodating the individualized approach to health coaching that has been successful in our previous research [67] and aids in building self-efficacy through gradually sitting less and building toward becoming more physically active. However, to facilitate more group connection in the future iteration of the program, we will provide participants with personalized resources where they can exercise for free, or at low cost with groups of people in their community or neighborhoods. These resources will be shared with participants who value group activity and who need a sense of community during their health coaching calls. Participants also expressed a desire to have some study contacts occur via teleconference platform, which will be accommodated in the larger trial by offering coaching calls via telephone or teleconference. Moreover, the study will create a newsletter that will be sent to all participants regularly highlighting study progress, status, team updates, and finally, the results of the study.

Following Consolidated Standards of Reporting Trials (CONSORT ) 2010 recommendations for pilot and feasibility studies [68], we present pre-post data on the proposed outcomes (objectively measured PA via activPAL and Fitbit), potential mechanisms of adherence, and other measures of interest to assess the feasibility of completion (for descriptive purposes). While PA pre- to posttest did not differ based on Fitbit, there was no true baseline period for the Fitbit as people started to use it immediately after receiving it. The activPAL was worn as an indicator of baseline PA and was worn prior to receipt of the Fitbit. We saw improvement in psychosocial mechanisms over the course of the study as well as some neurocognitive outcomes. However, given the small sample size, short duration, and lack of a comparison group, we cannot interpret these findings. As mentioned earlier, these measures were collected to assess the feasibility of completion, not for inferential purposes. The larger stage II trial will investigate the efficacy of the De Pie y a Movernos intervention on increasing PA, mechanisms of adherence, and potential effects on cognitive function.

This study is not without limitations. First, the pre-post pilot intervention was comprised of a small sample size of 10 participants and may not have been fully representative of the target community. Second, the duration of the intervention was short (3 weeks), which does not allow us to test the efficacy of the pilot intervention. That said, the goal of the pilot intervention was not to test efficacy, but rather to aid in the cultural adaptation process and determine the interventions’ feasibility and acceptability to inform a planned stage II trial. Third, the Fitbit and activPal measurement periods were nonoverlapping, thus not allowing for a true comparison between these 2 PA measurement modalities. There are several strengths of this study that should be highlighted. There are few existing studies on culturally adapting PA interventions for brain health in older Hispanics [69]. We involved a diverse CAB comprised of adults from the target population with several occupational and educational backgrounds, a fully bilingual and bicultural staff, and the development of the intervention based on established behavior change theory [40,41], which has been supported by previous studies conducted by the research team [67,70].

In the development and cultural adaptation of the De Pie y a Movernos intervention, we followed a community-engaged approach which led to a feasible and acceptable intervention with a 100% completion rate in our first iteration. These findings are consistent with previous studies showing good adherence and enjoyment of PA interventions that are culturally adapted and learn from participant feedback in the Hispanic community [33,69,71]. The CAB input was crucial to the development of a recruitment plan and recruitment materials, further highlighting the importance of community engagement in the design of research studies [32]. The combination of participants and CAB’s input informed the development of the culturally adapted De Pie y a Movernos intervention to be further tested in a large stage II RCT. The ultimate goal of the larger trial is to help older Hispanic adults increase their PA engagement to preserve their brain health and reduce dementia risk.

In conclusion, the De Pie y a Movernos intervention was deemed feasible and acceptable, meeting the necessary milestones to justify efficacy testing in a larger stage II PA trial. We highlight the importance of including the target community in intervention development from study conception. Using a community-engaged approach and obtaining participant feedback from a small pilot study allowed us to develop a feasible and acceptable intervention that was culturally adapted to address the specific concerns of the Hispanic community. In the next iteration of the study, we will implement the changes highlighted during the focus groups and test the De Pie y a Movernos intervention for efficacy to increase PA while continuing to obtain participant and CAB feedback for further refinement.

## Supplementary material

10.2196/65489 Multimedia Appendix 1 Final focus group discussion questions.

10.2196/65489 Multimedia Appendix 2 Final qualitative codebook.

10.2196/65489 Multimedia Appendix 3 Study satisfaction survey.

10.2196/65489 Multimedia Appendix 4 Summary of key themes and pilot program feedback from participant focus groups.

## Abbreviations

ADRD: Alzheimer disease and related dementias
CAB: community advisory board
CONSORT: Consolidated Standards of Reporting Trials
COREQ: Consolidated Criteria for Reporting Qualitative Research
IRB: institutional review board
PA: physical activity
RCT: randomized controlled trial
REDCap: Research Electronic Data Capture
