# Supplementary material for: Feasibility and Cultural Adaptation of a Community-Engaged Physical Activity Intervention for Hispanic Older Adults: Pilot Study
Source: JMIR Form Res. 2025 May 27;9:e65489. doi: 10.2196/65489 (PMC12154937; doi:10.2196/65489)
Supplement: Multimedia Appendix 1 [file formative-v9-e65489-s001.docx]

**Appendix 1**. Final Focus Group Discussion Questions

# Overall reﬂections and thoughts about the program

Great - Thank you for sharing your introductions!

- 1. So far, you’ve all shared some great things: *<facilitator reﬂect back to the group some of the things people shared in intros>.* Let’s keep building on what each of you shared so far and talk as a group. Are there any other parts of the De Pie Program that haven’t come up that were things you really liked or found helpful about the De Pie program?
     1. What did you like about that?
     2. Can you think of any ways you might have made that item/activity even more helpful or enjoyable?
  2. What about the opposite – were there any parts of the De Pie Program that you really didn’t like, were not helpful for you or you found particularly challenging?
     1. What didn’t you like about it?
     2. If you could change that item/activity, how would you make it more helpful/enjoyable?

Additional Prompts for these items if topics/components don’t come up organically:

- - - - What did you think about using the **Fitbit** device?
      - What did you think about working with your **health coach**?
      - What did you think about the **written materials from the study**? (e.g., the study ﬂyer, handouts, etc.) *Note: if prompting about study ﬂyer, show visual on the screen for feedback.*


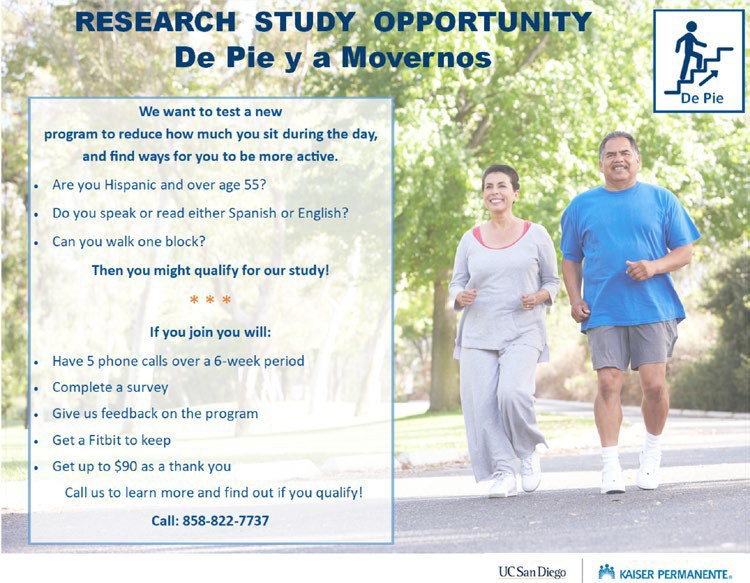


- - - - Did you ﬁnd it helpful to involve a **family/friend support person** or buddy during the program?
      - What did you think about wearing the **activPAL**, the small device that sticks to your thigh?
        - Instead of this device that sticks on your leg, would you have been willing to wear a small device that sits at your waist using a small elastic belt?
      - What did you think of the **cognitive assessments** you did on the phone?
      - What did you think of the **survey** you completed online?
      - How did **communicating with the study team** go for you? Was it easy to communicate with the team?
      - Was as it easy to understand what this research study was about?
      - Would more information about the importance of PA for preventing memory problems had been helpful?

# Study Logistics

1. How did you feel using phone calls to interact with your health coach? Would you have liked to receive some or all of your coaching and intervention content by text message instead of arranging phone calls?
2. We’re interested in understanding if using zoom for more study activities – like working with your health coach – would be something people might like. First, could you raise your hand if you already use Zoom and are comfortable with it? <*count responses>* Thanks. And how did you all like using the phone for study activities? Do you think you would you have preferred using Zoom or something similar?

# Program Structure & Content (Stairstep)

1. Were there any barriers that made it hard to participate in the study?
   1. What, if anything, made it challenging to meet the goals you set during the program?
   2. Did you ﬁnd ways to overcome or work around any of these barriers?
2. Were there any physical activities we didn’t highlight you would have like to see in materials (like dance, sport, play with grandchildren)?
3. What types of activity do you most like to do?
4. Are there any activities that you ﬁnd particularly meaningful culturally or to your community?
5. Are there any activities you really do NOT like doing?
6. The De Pie program is currently structured in a stair step framework. (***Show visual***)


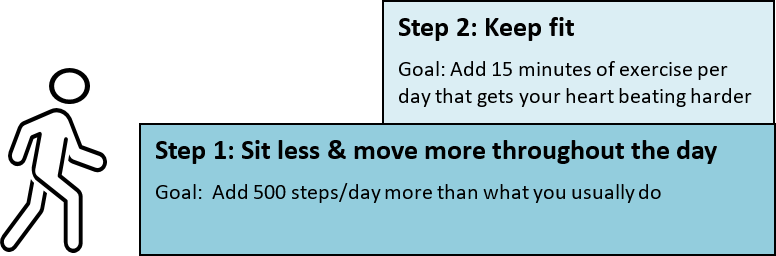


1. Did the two “steps” in the program make sense to you? Was the distinction between the two steps clear?
   1. Reminder: Step 1 focused on sitting less and adding small amounts of physical activity throughout the day (like getting up to do a lap around your home). Step 2 focused on adding more bouts of moderate activity or exercise in longer, dedicated bouts (like going for a walk). Was that a useful approach? Did you ﬁnd it useful to think about how you move on a typical day?
2. What did you ﬁnd confusing or not like about it?
3. What did you like or ﬁnd helpful about it?
4. To help us wind down our discussion today, I’d love to again go around the room and give each person an opportunity to share what motivated them to participate in the De Pie program and any ﬁnal thoughts about the De Pie program they haven’t yet had a chance to share. Like with our icebreaker, we’ll give each person 2-3 minutes. You’re welcome to say “pass” if you’d prefer not to share.
5. We’ve found that it can be helpful to future participants to hear and read stories or testimonials from people who have been in their shoes and done the program before. Would any of you be willing to share your story with the De Pie Program if we de-identify it? If so, raise your hand and
