# Supplementary material for: Feasibility and Cultural Adaptation of a Community-Engaged Physical Activity Intervention for Hispanic Older Adults: Pilot Study
Source: JMIR Form Res. 2025 May 27;9:e65489. doi: 10.2196/65489 (PMC12154937; doi:10.2196/65489)
Supplement: Multimedia Appendix 2 [file formative-v9-e65489-s002.docx]

**Appendix 2.** Final Qualitative Codebook

# De Pie Pilot – Focus Group Thematic Codebook

**Study Feedback**

Measurement Devices (MD)

- Positive activPAL (MD-APP): positive comments about the activPAL device; ﬁnding it easy to wear, unobtrusive, etc. or liking the feedback/data from the device
- Negative activPAL (MD-APN): negative comments about the activPAL device; ﬁnding it diﬃcult to wear, annoying, uncomfortable, etc. or not liking or ﬁnding helpful any feedback/data from the device
- Positive Fitbit (MD-FBP): positive comments about the Fitbit device; ﬁnding it easy to wear, unobtrusive, etc. or liking the feedback/data from the device
- Negative Fitbit (MD-FBN): negative comments about the Fitbit device; ﬁnding it diﬃcult to wear, annoying, uncomfortable, etc. or not liking or ﬁnding helpful any feedback/data from the device
- Positive Actigraph (MD-AGP): positive comments about the Actigraph device; believing it would be easy to wear, unobtrusive, etc.
- Negative Actigraph (MD-AGN): negative comments about the ACtigraph device; believing it would be diﬃcult to wear, annoying, uncomfortable, etc.

Written Materials (M)

- Positive Flyer (M-FP): positive comments about the ﬂyer, things participants mentioned that they like in the current ﬂyer or would want us to keep in future versions
- Flyer Improvements (M-FI): negative comments about the ﬂyer or suggestions for improving it in future versions
- Positive workbook (M-WP): positive comments about the study workbook, things participants mentioned that they like in the current workbook or would want us to keep in future versions
- Workbook Improvements (M-WI): negative comments about the workbook or suggestions for improving it in future versions
- Positive survey (M-SP): positive comments about the study surveys or their administration, things participants mentioned that they like or found easy to complete in the current survey or would want us to keep in future versions
- Survey Improvements (M-SI): negative comments about the study surveys or their administration or suggestions for improving it in future versions
- Positive Device Instructions (M-DP): positive comments about all written materials supporting the Fitbit and/or activPAL, comments about ﬁnding these materials easy to follow and supporting their successful use of the devices
- Device Instructions Improvements (M-DI): suggestions for improvement for all written materials supporting the Fitbit and/or activPAL, comments about not ﬁnding these materials easy to follow or not supporting their successful use of the devices
- Positive Other Study Materials (M-OP): positive comments about any other written study materials (e.g., study timeline, letters, etc.), comments about ﬁnding these materials easy to follow and supporting their successful engagement in study activities
- Other Study Materials Improvements (M-OI): suggestions for improvement for any other written study materials (e.g., study timeline, letters, etc.), comments about not ﬁnding these materials easy to follow or not supporting their successful engagement in study activities

Health Coach (HC)

- Positive Coaching (HC-P): positive comments about their health coach or the experience of receiving health coaching; ﬁnding helpful, motivating, supportive, etc.; comments about coach providing additional information or support that was helpful or empowering
- Coaching Improvements (HC-I): suggestions for improving the coaching experience; changes that would help make coaching more eﬀective, supportive, or engaging; feeling uncomfortable with their coach or the coaching approach.

Cognitive Assessments (CA)

- Positive Cognitive Assessments (CA-P): positive comments about the experience of completing cognitive assessments by phone; ﬁnding it easy to understand and engage with by phone, feeling comfortable with the process and the assessor
- Cognitive Assessment Improvements (CA-I): suggestions for improving the cognitive assessment experience; changes that would help the remote cognitive assessment process easier to engage with or complete; feeling uncomfortable with the process or the assessor

Communication (C)

- Positive Communication (C-P): positive comments about the experience of communicating with the study or study team members; ﬁnding the frequency, mode and ease of communication to be good
- Communication Improvements (C-I): suggestions for improving the experience of communicating with the study or study team members; comments about inappropriate frequency or mode of communication, challenges reaching the study or making appointment times
- Video Conference Platform (C-V): comments about use of a video conference platform (e.g. zoom, skype, Teams, etc.) for the intervention or other study activities
- Email (C-E): comment about use of email for administering intervention content or other study activities
- Text (C-T): comment about use of text messaging for administering intervention content or other study activities

Program Structure & Purpose (P)

- Positive Purpose (P-PP): comments that reﬂect understanding about the goal of the study being to increase physical activity/exercise to promote better cognitive health and aging, include comments about preventing dementia or Alzheimer’s disease and comments about exercise to improve cardiovascular or general health (including mental health)
- Negative Purpose (P-NP): comments that reﬂect the participant did not understand the long- term goal of the study being about improving cognitive health
- Positive Structure (P-PS): comments that reﬂect understanding the 2-step structure of the study and understanding that sitting less is distinct from engaging in more regular structured bouts of exercise
- Negative Structure (P-NS): comments that reﬂect participant did not understand the 2-step structure of the study and had diﬃculty understanding that sitting less was distinct from engaging in more regular structured bouts of exercise

Cultural Meaning (CU)

- Positive Cultural Meaning (CU-P): positive comments about how the program was meaningful to and representative of wants and needs of the Latino/Hispanic community in the San Diego area
- Cultural Meaning Improvements (CU-I): suggestions for making the program more meaningful or culturally appropriate/engaging to the people and needs of the Latino/Hispanic community in the San Diego area

# Physical Activity & Sedentary Behavior Change

Behavior Tags

- Physical Activity (PA): use to tag comments that are explicitly discussing a facilitator or barrier to PA (being more active, exercising, walking, etc.) as opposed to sitting less
- Sitting (SB): use to tag comments that are explicitly discussing a facilitator or barrier to sitting less/reducing sedentary behavior (breaking up sitting periods, reducing time in sitting activities, etc.) as opposed to physical activity/exercise

Facilitators (F)

- Awareness(F-A): having an increased awareness of their sitting and physical activity levels (e.g. from Fitbit or activPAL feedback) and the need to sit less and move more is motivating. Does not require that participant has taken action to change habits, just expressing new motivation.
- Fitbit (F-F): used study-supplied Fitbit Inspire band to prompt sitting breaks, track steps, or otherwise encourage less sitting and more movement and/or getting more active
- Habits (F-H): having built new habits around standing more/sitting less or new physical activities that are now part of daily routine. Can include action-oriented internal cues or external cues (including rearranging home environment, cue cards) that help them keep their habit of sitting less/being more active.
- Social Support (F-SS): Social support from family or friends to do physical activities or sit less makes changing these habits easier; include comments about desire for/interest in engaging with other study participants to encourage more activity
- Enjoyment (F-E): Enjoy certain types of exercise or other physical activities (including housework, playing with grandkids, or other active daily living tasks) that make engaging in PA fun and

something they want to do; can include ﬁnding new enjoyment in an activity they just tried or haven’t done in a long time.

- Self-Eﬃcacy (F-SE): Existing or new belief that participant is able to do what’s needed to be more active
- Accountability (F-AC): accountability through coaching makes habits and goals easier to maintain, encouragement through the health coach, knowing their activity is being monitored by coach/study; spreading awareness to others and holding them accountable for sitting less
- Mental Health (F-MH): noticing improvements in mental health due to increased activity and engagement with the program, these noted improvements encourage ppt to continue with increased activity.
- Motivation to Participate (F-M): explicit comments about a motivation to participate in the study; descriptions of how that motivation helped them change behavior (sit less, move more) in the study
- Other facilitators (F-O): And other facilitators mentioned not captured above

Barriers (B)

- Health problems/changes (B-H): Changes in overall health status or other competing health concerns have made changes in sitting and physical activity diﬃcult or caused backtracking; chronic conditions making it diﬃcult
- Fitbit (B-F): did not use study Fitbit Inspire or report ﬁnding it troublesome, annoying or not helpful, the device had problems, or otherwise didn’t like the Fitbit
- Habits (B-HB): having ingrained habits of sitting and/or not exercising make it diﬃcult to sit less or start a new physical activity routine (e.g. sitting during certain activities, etc.)
- Lack of Social Support (B-SS): lack of social support from family or friends to do physical activities or sit less makes changing these habits diﬃcult
- Enjoyment (B-E): Not enjoying physical activities or particularly enjoying sedentary activities that make it hard for participant to want to engage in more activity.
- Self-Eﬃcacy (B-SE): Existing or new belief that participant is NOT able to do what’s needed to be more active for any reason.
- Lack of Accountability (B-A): Loss of accountability when coaching ends makes new habits and goals diﬃcult to maintain
- Time Constraints (B-T): Busy schedules, lack of time, or other time constraints making participation in study activities or engaging in sitting less/moving more diﬃcult
- Weather (B-W): Diﬃculty engaging in physical activity or sitting less because of weather conditions (heat, rain, etc.)
- Other barriers (B-O): Other barriers like retirement or other transitions or life events
