# Supplementary material for: Feasibility and Cultural Adaptation of a Community-Engaged Physical Activity Intervention for Hispanic Older Adults: Pilot Study
Source: JMIR Form Res. 2025 May 27;9:e65489. doi: 10.2196/65489 (PMC12154937; doi:10.2196/65489)
Supplement: Multimedia Appendix 3 [file formative-v9-e65489-s003.pdf]

Appendix 3. Study Satisfaction Survey

Thoughts about the De Pie Program

The following questions ask for your thoughts about the De Pie y a Movernos program.

For each component of the program listed below, please choose the response option below to indicate how satisfied you were with it.

	Strongly Disagree	Disagree	Neutral	Agree	Strongly Agree
**1.** There were too many phone calls	1	2	3	4	5
**2.** The study consent process was easy to follow and convenient.	1	2	3	4	5
**3.** The Fitbit device was helpful and easy to use.	1	2	3	4	5
**4.** The activPAL device (thigh) was comfortable and easy to wear.	1	2	3	4	5
**5.** Instructions for the cognitive tests were easy to understand.	1	2	3	4	5
**6.** I liked that I didn't have to travel anywhere for study activities and could participate by phone and mail.	1	2	3	4	5
**7.** The length of study phone calls was reasonable.	1	2	3	4	5
**8.** Study phone calls were easy to hear.	1	2	3	4	5
**9.** I would be interested in doing study visits using Zoom.	1	2	3	4	5
**10.** Working with my health coach was helpful for setting and meeting my goals.	1	2	3	4	5
**11.** This program fit well with my family and daily life.	1	2	3	4	5
**12.** The written handouts were easy to understand and helpful.	1	2	3	4	5
**13.** I liked the study's focus on sitting less and moving more.	1	2	3	4	5
**14.** The materials in this study were meaningful to me and my life.	1	2	3	4	5
**15.** The examples and activity suggestions in this study made sense for my life.	1	2	3	4	5

**16.** It was easy to communicate with the study.	1	2	3	4	5
**17.** Overall study instructions were easy to understand and follow.	1	2	3	4	5
**18.** Overall, I liked the study and found it helpful.	1	2	3	4	5
