# Supplementary material for: Feasibility and Cultural Adaptation of a Community-Engaged Physical Activity Intervention for Hispanic Older Adults: Pilot Study
Source: JMIR Form Res. 2025 May 27;9:e65489. doi: 10.2196/65489 (PMC12154937; doi:10.2196/65489)
Supplement: Multimedia Appendix 4 [file formative-v9-e65489-s004.pdf]

Appendix 4. Summary of key themes and pilot program feedback from participant focus groups.

Domain	Theme	Examples	Supporting Quotations
**Study Feedback**			
Activity Devices	Fitbit	**Positives**:<br>• Informative about current activity levels<br>• Motivating to break up sitting and move more and improve other healthy habits (sleep, hydration)<br>• Easy and enjoyable to use and incorporate into daily life/routines	"…it's something basic for me, it has become part of my life, and I'm not going to give it up. I always wear it here." – P15<br>"Yes, it helped me a lot. Every time my little watch rang, I knew I had to get up and keep going." – P19<br>"It helped me a lot because I realized that I wasn't doing any activity." – P17
		**Negatives**:<br>• Challenging for some to set up and install, needed support<br>• Faced connection issues between band and App<br>• Band causes skin irritation	"The honest truth for me is that it was really hard for me to download Fitbit app, and if it hadn't been for my daughter, I wouldn't have been able to download it." – P3<br>"In my case, because it irritates my skin a lot. So the Fitbit irritated me a lot. I don't know why, if my skin is sensitive or what?" – P1<br>"But the same with this device, in the end both P7 and I were disconnected from the app" – P8
	activPAL	**Positives**:<br>• Worked as expected from materials, no problems<br>• Easy to wear and didn't irritate or interfere with daily tasks<br>• Some preferred a device on the leg vs. waist	"Everything was perfect for me. It didn't irritate me at all. I mean, for me it was perfect for seven days." – P15<br>"No, it's much better for me on my leg…Because on your leg it's... one, it's hidden, it doesn't bother you as much. Two, it's more accessible, more comfortable." – P17
		**Negatives**:<br>• Device failed to record, effort to wear was wasted<br>• Irritating to skin and/or difficult to adhere, particularly in warm climate	"Because when the light didn't flash, that is, the... It wasn't activated...So I had it for several days for nothing. And it was irritating my skin…" – P1<br>"And besides, it's very hot here where I live. Immediately after an hour, my entire leg was red where the device was." – P3
Study Materials	Workbook	**Positives**:<br>• Easy to navigate and content is clear and understandable<br>• Helpful ideas and suggestions	"They're the little sheets, they come stapled together, right? They're not separated, and it's very good for me. All the instructions and everything are very clear" – P15<br>"About the exercises, I also liked them. I liked the ideas they gave me." – P2
		**Improvements** (code volume: 0):<br>• No specific suggestions voiced	N/A
	Written Device Instructions	**Positives**:<br>• Found device instructions (activPAL or Fitbit) easy to use, especially with phone support from team.	"It was easy with the Fitbit because LML was with me giving me clear instructions, and with the device I put on my leg it was also easy. I did it quickly. I understood it." – P17
		**Improvements**:<br>• Written instructions unclear or insufficient to support successful device set up or successful wear<br>• Instructional videos may be helpful<br>• Not clear where to begin/first step, want a cover sheet<br>• Not clear if activPAL is continuously sending data	"Possibly with videos, yes. But the instructions were very brief for me, and I couldn't do it by myself." – P3<br>"I got confused when I opened the package. As soon as I opened it, I said, "Oh, I have to download the app." Then I went through the steps, but no." – P20
		**Positives**:	"The materials are also easy to understand. And what we didn't understand, as she said, we had the confidence to ask." – P2

	Other Study Materials	• Generally found materials clear and easy to understand<br>• Appreciated team roster with photos<br>• Materials made program goals clear and easy to connect with	“The moment I received the package, I started, well, to quickly read and see. And I said, ‘Wow, this program is very important to me, and let's see what it’s going to bring us.’” – P15<br>“I really liked the package with all the photos of all you guys. When I talked to you on the phone, I already knew who I was talking to.” – P20
		**Improvements**:<br>• Cover sheet clearly stating where to begin would be helpful<br>• Separate sheets easy to confuse/get out of order; suggest putting all materials in a folder/binder for clearer order<br>• When possible, present same info in multiple ways to accommodate different learning styles<br>• More information about the relationship between PA and cognitive health would be helpful<br>• More community resources, options for in-person group exercise classes (e.g., Zumba) in community desired	“I would have liked, for example, and this is an idea, for example, of something, a page where it says, ‘What to do as soon as you receive this package. Number one, number two, number three.’” – P20<br>“If you could put it in a folder, I mean, since it all comes together…If it could be put in a folder, and then we could see the follow-up page by page.” – P19<br>“But we have to take into account, in my opinion, that we have different learning styles, reading styles, styles...” – P1<br>“We know we need to do it, but sometimes we put it aside. I'm more of a visual person and I always like to leave things like this…in sight and it reminds me that I have to do it, or it catches my attention again. If it’s a brochure [about PA and cognitive health], I'm interested in reviewing it again.” – P2<br>“I want to do a little bit of what we were talking about regarding the group activity. I like it very much because I’m a community leader, and the group activity… you get feedback from each person, from each story, and it's not just going to take the class.” – P17
Study Activities	Health Coaching	**Positives**:<br>• Helped set goals, generate ideas, break down content into achievable pieces<br>• Provided accountability and follow-up on goals<br>• Provided clear instructions and patient support about study, answered participant questions	“It challenged me more because I knew that you guys were going to call me to see how I did, what I did, and what I didn’t do.” – P1<br>“He also helped me fill all this out, the activities that I was doing and what I wanted to do in the future and try to carry out, right?” – P15<br>“It was incredible the way he answered, the way he gave me instructions, guided me, answered my questions.” – P20<br>“I loved it. I loved it because we walked hand-in-hand.” –P17
		**Improvements**:<br>• Would have preferred in-person coaching<br>• Some suggestions, ideas from coach not a good fit	“But I personally like to be in person at activities like this, trainings or workshops, right?” – P2<br>“So he would tell me, "And walk up the stairs,” because I live on the fourth floor. "No, I want to dance.” I mean, that doesn't motivate me.” – P1
	Communication	**Positives**:<br>• Clear in interactions that team knows and cares about Latino culture<br>• On time for scheduled calls<br>• Provided follow-up and support with questions & challenges throughout study<br>• Staff were supportive and reassuring<br>• Some appreciated phone as lower tech primary communication option	“When I communicated with each one of you, it was clear that you know a lot about the culture, and that’s very important in making a connection with the community.” – P20<br>“Yes, I also thought the communication was very good. LML was very patient, and with all his patience he helped me on several occasions. And the cognitive aspect as well.” – P19<br>“when they were asking me over the phone, like right now, I had no problems. With my computer [using Zoom], I couldn’t do it.” – P19
		**Improvements**:	“It’s preferable for people to have more options and to choose that option. Because if you guys had asked me from the beginning, "P1,

		• Provide options for communication mode when possible<br>• Many preferred a Zoom option for some activities (e.g., coaching, cognitive assessment)<br>• Offering in-person meet up options with other participants desired by some<br>(*Notably, email and text communication modes were not discussed or highlighted by participants as positive or negative options.)*	do you want it in person or over the phone?" I would have said, "Zoom, or in person."" – P1<br>"Although, as my colleagues said, you have to have a few more options. Because we're all different, and we all take in everything differently. So there needs to be options for everyone." – P15
Program Structure & Purpose	Purpose of intervention	**Positives**:<br>• Found program goal and purpose clear<br>• Program goal resonated as important to participants' lives	"So I think this program…was for the elderly… and within the Latino community, knowing that there are high numbers of dementia among Latino and African American people…I think that's the purpose of maybe reducing the risks of disease, not just dementia, but any other disease, right?"–P1<br>"And the entire program was very satisfying for me. It was what I imagined, what I expected." – P15
	Cultural Meaning	**Positives**:<br>• Cognition/dementia and physical activity focus of study important to Latino community<br>• Study offered in Spanish<br>• Study team part of/knows a lot about the Latino community<br>• Focus of study specific to older Latinos made participants feel seen/appreciated as a community	"Above all, I really liked that it was in Spanish." – P2<br>"Personally, I basically want to thank you for this study, this information that is so important for the community."- P2<br>"it was clear that you know a lot about the culture, and that's very important in making a connection with the community." – P20<br>"…many Latino people don't understand or don't, sorry, they haven't had much of that important health experience about exercise that affects our mental health, I mean, we can provide education about depression or more diseases…" - P1<br>"I said, "Wow, someone is concerned about us Latinos, about us old people, the elderly Latinos." – P15<br>"And thanks to you guys who are helping Latinos more than anything else. Because there aren't many programs that support Latinos more than anything else." -P3
		**Improvements**:<br>• Including more opportunities to connect with community, exercise in community would be meaningful<br>• Opportunities to connect with other study participants nearby would be meaningful	"But there should be that connection because as a community worker, as a community leader, I always try to look for that connection between me and my community." – P17<br>"But, as our colleague mentioned, they're also experiences that we can share and learn more from each other as well. Or what one of us was concerned about, to say, "Well, I'm not the only one who has it. There's someone else who has it, but how does she look at it? It helps me, I get it." And sharing those experiences or those fears, right?" – P2
Barriers to PA		▪ Lack of awareness: Minimal or poor understanding of how physical activity impacts cognitive and physical health.<br>▪ Lack of Self-efficacy: intrinsic belief in the inability to do PA<br>▪ Health changes or problems<br>▪ Lack of enjoyment, not enjoying PA or study activities<br>▪ Lack of social support from friends/family to engage in PA<br>▪ Habits: having ingrained habits of sitting and/or not exercising	"Many Latino people don't understand, they haven't had much of that important health experience about exercise that affects our mental health, they're more focused on taking care of the kids, taking care of the husband, and they don't focus a lot on taking care of themselves" – P1.<br>"What I wouldn't do is run because I'm not in good shape or... I don't know, I can't run" – P2.

		▪ Fitbit: finding the device annoying or troublesome<br>▪ Environment/Weather<br>▪ Time constraints	"But even though I was motivated by the study to do what I was asked to do, my migraines and my stress and everything that accumulated in me didn't let me" – P19<br>"But I'm not one to go to the gym or anything like that" P7<br>"A lot of friends, even though they're nice people, they don't know about health, or they don't have that ability to really motivate you. They use things like, "Oh, no, what are you losing weight for, or what's this for?" – P1<br>"My job is fixing clothes at home. I sit at the machine all day" – P7.<br>"I'm really into soap operas. I love soap operas, television, I'm retired. I mean, I go out a lot, but I also watch a lot of TV" – P20.<br>"In my opinion, the biggest obstacle is the weather. It's very hot here, and you can't go for a walk" P3<br>"It doesn't matter what's next to us, because we always say, I have chores to do, I have my house, my children, my grandchildren, my husband…" - P17
Facilitators to PA		▪ Awareness: Increased awareness of current PA and/or cognitive function. Understanding how physical activity impacts cognitive and physical health.<br>▪ Accountability: Sense of commitment to study obligations making goals and habits easier to maintain in response to coaching, activity monitoring, etc.<br>▪ Self-efficacy: Realization (existing or new) in their ability to increase PA and the need to prioritize self-care<br>▪ Enjoyment: Finding a new PA or resuming a previous activity not performed for a long time that is enjoyable<br>▪ Social Support from family/friends or support from the study<br>▪ Habits: building new habits around PA, making it routine<br>▪ Fitbit<br>▪ Physical and Mental Health improvements	"So, this personally gave me the opportunity to realize that it was necessary to look for movements, to go back again to what I longed for, and I still weep to go jogging" P8.<br>"But the program makes you aware, it motivates you along with the importance of keeping in shape and moving more. And so, you have to look for it" - P3.<br>"Yes, it helped me a lot, quite a lot, the way made me see things, how I could, little by little, make changes" – P19.<br>"When they explain to you that you can exercise by starting small, at your own pace, and when you feel pain, you stop, well, I looked at it differently" - P2.<br>"I learned with him to realize that I love myself...", "I have to take care of myself and to seek my wellbeing for myself, not for other people". - P17<br>"I'm motivated by dancing by music. And I don't drink, I dance without liquor" P1.<br>"That's my goal, to go back to jogging I used to do before because I loved it" – P8.<br>"That's what motivates me the most, that I have someone. And that person who motivated me to enter is my only friend who helps me to keep going" – P19.<br>"I used to be one of those who looked for the closest parking spot so I wouldn't walk. Now I don't mind being farther away" - P2.<br>"I really liked it. Do you know why? Because I feel pressured because it tells me when my time is about to run out and I haven't completed my steps" - P15<br>"I also really liked Fitbit. I don't leave it behind, and it reminds me to drink water and that more than anything I have to move" – P3.<br>"When I started to increase my steps every day, I saw that I felt better, I slept better, I had more energy, I felt good about myself, I felt a little bit more... I like the way I feel" – P20.<br>"You feel the difference in your state of mind, in your state of health in general" - P1.
